# Aqueous Biphasic Systems Based on Tetrabutylammonium Bromide for Extraction and Determination of Azorubine, Allura Red, Sunset Yellow, Tartrazine and Fast Green in Food Samples

**DOI:** 10.3390/molecules30244769

**Published:** 2025-12-13

**Authors:** Svetlana V. Smirnova, Anastasia V. Gorbovskaia, Yulia S. Vershinina, Vladimir V. Apyari, Mikhail A. Proskurnin

**Affiliations:** 1 Chemistry Department, Lomonosov Moscow State University, Leninskie Gory 1, Moscow 119991, Russia; gorbovskaya_av@mail.ru (A.V.G.); yu.vrshn@gmail.com (Y.S.V.); apyari@mail.ru (V.V.A.); proskurnin@gmail.com (M.A.P.); 2 Federal State Budgetary Institution of Science Institute of African Studies, Russian Academy of Sciences, St. Spiridonovka, 30/1, Moscow 123001, Russia

**Keywords:** aqueous biphasic systems, aqueous two-phase systems, green liquid–liquid microextraction, tetrabutylammonium bromide, food dyes, 1-octanol–water distribution coefficients

## Abstract

Aqueous biphasic systems (ABSs) based on tetrabutylammonium bromide (TBABr) and potassium thiocyanate or citrate (K_3_Cit) were proposed as “green” tools for liquid–liquid microextraction of Azorubine, Allura Red, Sunset Yellow, Tartrazine and Fast Green followed by spectrophotometric determination. The dye extraction into the water-saturated tetrabutylammonium thiocyanate phase, which separates from water when mixing aqueous solutions of TBABr and KSCN, depends on the hydrophobicity of dyes. Only Azorubine is extracted quantitatively at HCl concentration ≥ 0.05 mol L^−1^, with an equimolar TBABr/KSCN ratio and total concentration of 0.4 mol L^−1^ in less than 1 min. To estimate the hydrophobicity and identify factors affecting the distribution of dyes in ABSs, the experimental 1-octanol-water distribution coefficients of the dyes were determined. In contrast, all the dyes studied are quantitatively extracted into the TBABr–K_3_Cit–H_2_O ABS regardless of their hydrophobicity. The effects of pH, concentration of phase-forming components, extraction/centrifugation time and other factors were investigated for both ABSs. The linearity ranges and detection limits were 0.05–2.60 mg L^−1^ and 0.006–0.02 mg L^−1^, respectively. The proposed procedures were applied for the determination of dyes in food samples.

## 1. Introduction

Modern commercial food production is difficult to imagine without the use of food additives. One of their most common kinds is synthetic food dyes utilized to preserve the original coloring of food products, which they may lose as a result of cooking technology, heat treatment and storage [1]. There are known cases of food adulteration in order to imitate the increased nutritional value and consumer properties of the product by adding dyes or flavors using authorized and unauthorized additives [2]. Ease of production, low cost, accessibility, variety of saturated colors, high intensity, stability and uniformity of coloring with synthetic food dyes ensure the growing commercial demand and their predominance in the market compared to natural dyes and pigments [3,4]. Anionic dyes, usually bearing the -SO_3_^−^ groups (azo- and triphenylmethane dyes), are the most common synthetic dyes used for manufacturing a wide range of beverages, juices, confectionery and other food products [5,6]. They have the desired color shades and improved solubility [7]. At the same time, over-intake of them via foodstuffs can lead to food intoxication as a result of their metabolism [8]. This can cause severe health problems such as asthma, allergic reactions, weakening of the immune system, and also affect hyperactivity in children [4,9]. Thus, the Acceptable Daily Intake (ADI) limits of Allura Red, Azorubine, Fast Green FCF, Sunset Yellow, and Tartrazine have been set by the World Health Organization at 7.0, 4.0, 25.0, 2.5 and 7.5 mg/kg bw/day, respectively [10]. Moreover, photodegradation of dyes can result in the formation of potentially toxic substances [11,12].

In this context, the development of efficient analytical methods of monitoring and quantitative determination of dyes in food are required. However, the complexity and variety of food matrices can significantly reduce the accuracy, even when using highly sensitive and selective methods such as high-performance liquid chromatography, reversed phase ion-pair chromatography, thin layer chromatography, capillary electrophoresis, liquid chromatography/tandem mass-spectrometry, and fluorescence spectrometry [13,14,15,16,17]. Therefore, it is essential to employ optimal pretreatment procedures to minimize matrix effect by effectively separating target analytes from interfering components. In addition to the requirements for sensitivity and accuracy, the modern determination method should also be cost-effective, affordable, and straightforward to implement [18]. However, most of these methods require higher operating and maintenance costs. Sample preparation, which includes separation and preconcentration steps, often retains an advantage of spectrophotometric determination, having simplicity, cheapness, ease of operation, and accessibility in many laboratories.

Currently, liquid–liquid extraction is often preferred, partly because of the simplicity of use, capacity to produce high extraction efficiency and high enrichment factors in a short time and, most importantly, because of the wide range of different environmentally friendly extraction systems capable of providing high analytical performance [3,13]. Much attention is paid to very popular extraction systems based on ionic liquids (ILs) [19,20], deep eutectic solvents (DESs) [21,22,23,24,25,26], switchable hydrophobic solvents [27], hydrophilic solvents [28], supramolecular solvents [29], ion-pair solvents [30], surfactants [5,31], and aqueous biphasic systems (ABSs) [32,33,34,35,36,37,38,39,40].

The attractiveness of ABSs for sample preparation is related to their distinctive feature, which is the high water content in both immiscible phases [34]. For the extraction of hydrophilic compounds that are highly soluble in water, this can be a key factor in ensuring high extraction efficiency. In addition, these systems have significant potential for dye preconcentration due to their versatility. ABSs are formed by mixing various incompatible water-soluble compounds such as salts, polymers, surfactants, ILs, sugars, amino acids and alcohols in an aqueous medium. When the concentration exceeds a critical value, the liquid–liquid separation is observed [32,35].

This study proposes ABSs based on inexpensive tetrabutylammonium bromide (TBABr), which offers low volatility and unique phase-forming capabilities [34]. TBABr-based ABSs enable rapid phase separation with low viscosity and accommodate wide concentration ranges for system formation. The high phase volume ratio makes these systems particularly suitable for preconcentration of dyes. We demonstrate that salt-salt ABSs based on TBABr can exhibit varying extraction capacities for dyes, depending on the nature of the second salt.

The nature of the ABS components determines the differences in the composition and properties of the two phases, as well as the factors and mechanisms driving the separation. The second salt can either act as a salting-out agent, promoting the separation of a TBABr-rich phase, or become part of the extraction phase itself. Potassium citrate (K_3_Cit) has a high salting-out capacity and can be used to salt out TBABr. The phase enriched with TBABr has high extraction efficiency with respect to anionic dyes, regardless of their hydrophobicity. Moreover, potassium citrate is biodegradable, non-toxic, and readily available, making it a promising compound for sustainable food and environmental applications. The addition of KSCN to an aqueous TBABr solution leads to the separation of water-saturated tetrabutylammonium thiocyanate (TBASCN). This approach is particularly important for developing more economical and environmentally friendly systems by reducing overall salt concentration. Both precursors are significantly cheaper and more readily available compared to pre-synthesized TBASCN. Although the ability of TBASCN to form a liquid–liquid system with water was first reported in [41], this promising system has been overlooked for extraction until now. The efficiency of dye extraction into the TBASCN-rich phase of the TBABr–KSCN–H_2_O ABS depends on the dye’s hydrophobicity. To estimate the hydrophobicity and identify factors affecting the distribution of dyes in ABSs, the experimental 1-octanol-water distribution coefficients (Log P) for the studied dyes were determined. Furthermore, understanding the partitioning behavior of dyes and other pollutants is important for optimizing purification and extraction processes, as well as for assessing their environmental impact [42].

Thus, this work is dedicated to developing and evaluating two novel TBABr-based ABSs for the efficient extraction of hydrophilic sulfonated dyes. We investigated how two distinct approaches to ABS formation—salting-out with K_3_Cit versus metathesis with KSCN—affect the extraction efficiency and selectivity, and how the dye distribution in these systems correlates with its hydrophobicity.

To the best of our knowledge, this is the first study to employ a novel TBABr–KSCN–H_2_O ABS for preconcentration without the addition of salting-out agents. Both ABSs were investigated for liquid–liquid microextraction of anionic dyes Allura Red, Azorubine, Fast Green, Sunset Yellow, and Tartrazine from food samples prior to spectrophotometric determination. The proposed systems offer several advantages, including short extraction time, rapid phase separation, low viscosity, lower cost and the availability of their components.

## 2. Results and Discussions

Three sets of experiments were carried out to explore the potential of salt-salt ABSs for extraction and preconcentration of dyes. In the first set, the novel phase diagram for the TBABr–KSCN–H_2_O system was constructed and compared with the phase diagram of TBABr–K_3_Cit–H_2_O. In the second part, the distribution of dyes in 1-octanol–water system was studied to compare with their extraction in ABSs. Finally, the conditions for quantitative extraction and analytical performance of the proposed procedures were evaluated.

### 2.1. Formation and Properties of Tetrabutylammonium-Based ABSs

**Figure 1:**
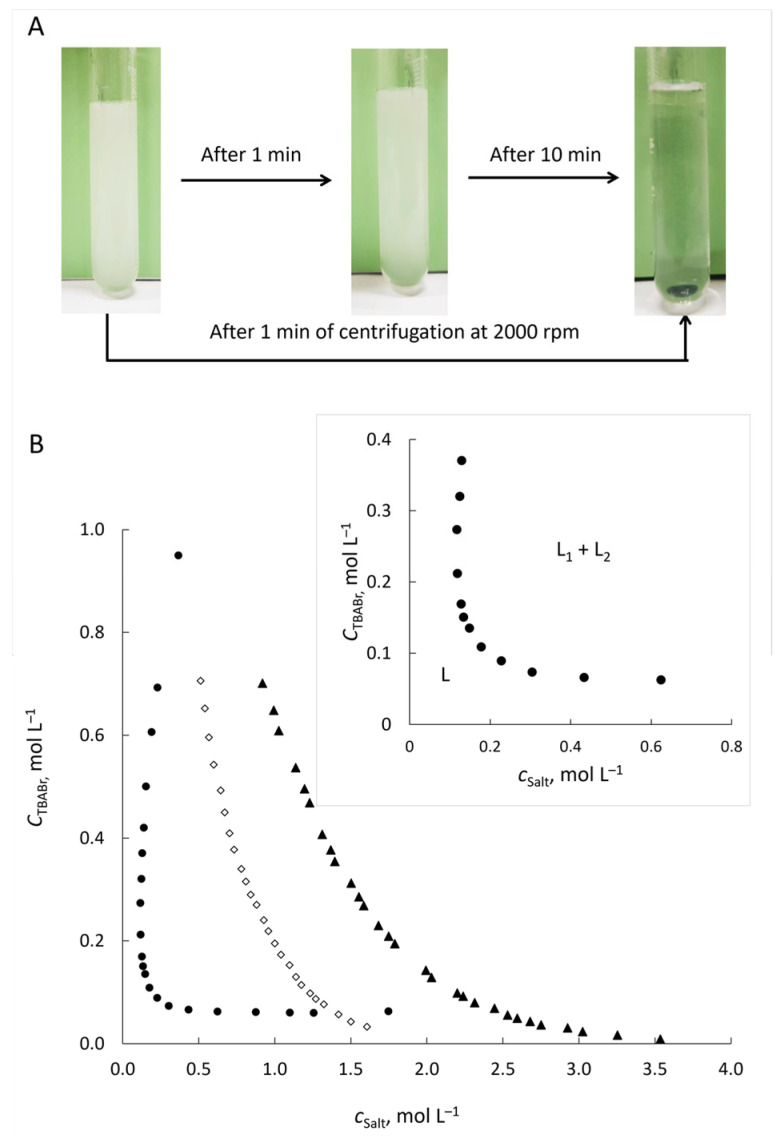
Phase separation after mixing of aqueous solutions of TBABr (0.3 mol L^−1^) and KSCN (0.3 mol L^−1^) (**A**). Phase diagram for tetrabutylammonium-based ABSs at 22 ± 2 °C (**B**). ABS formed with (●) KSCN, (◊) K_3_Cit, or (▲) (NH_4_)_2_SO_4_. The data for TBABr–(NH_4_)_2_SO_4_–H_2_O ABS is taken from [47].

The novel ABS consisted of two salts present in comparable quantities: the organic salt TBABr and the inorganic salt KSCN. The phase-forming components of the studied system are highly soluble in water [43,44]. Therefore, it is more convenient to obtain the ABS by mixing aqueous solutions of TBABr and KSCN. In addition, it was found that neither the order nor the method (using solid salts or pre-made aqueous solution) of mixing the phase-forming components affected the system formation process or the dye extraction efficiency. Preliminary experiments indicated that mixing of aqueous solutions of TBABr and KSCN in an equimolar ratio at the total concentration of salts (*c*_t_) above 0.28 mol L^−1^ leads to the separation of the system into two immiscible liquid phases. The phase separation occurs spontaneously and rapidly (within 10 min, Figure 1A). The resulting phases are transparent and highly mobile liquids.

The phase diagram for the TBABr–KSCN–H_2_O system (Figure 1B) shows the range of total concentrations of TBABr and KSCN at which phase separation occurs. The homogeneous (single liquid phase) and heterogeneous (two immiscible liquid phases) regions are located below and above the binodal curve, respectively. A distinctive feature of the TBABr–KSCN–H_2_O system is its wide biphasic region, which forms at comparable, relatively low salt concentrations—unlike the high concentrations of conventional salting-out agents (K_3_Cit, (NH_4_)_2_SO_4_) required for TBABr-based ABSs.

As is known, the solubility of TBABr in water decreases in the presence of inorganic salts at high concentrations, which leads to the separation of the hydrophobic liquid phase enriched with TBABr. The salting-out of TBABr occurs due to the preferential hydration of the high charge density salt over TBABr, as well as a combination of electronic repulsion and an enhancement of the hydrophobic effect, followed by solutes aggregation and then their separation from the aqueous phase [45,46]. The bromide ion loses in competition with ions with a higher charge density, that is, capable of stronger interactions with water, for the formation of a hydrate complex. The concentration of the salting-out agent can be several orders of magnitude higher than the concentration of TBABr, especially for very dilute TBABr solutions [45,47]. This is well demonstrated in Figure 1 by the curves for TBABr-K_3_Cit-H_2_O and TBABr-(NH_4_)_2_SO_4_-H_2_O systems. The formation of ABS at a TBABr concentration of 0.03 mol L^−1^ occurs in the presence of 1.6 mol L^−1^ of K_3_Cit or 2.9 mol L^−1^ of (NH_4_)_2_SO_4_, consistent with the trend that more highly charged anions are more effective at salting out TBABr [45].

The ability of an ion to promote phase separation in ABS is usually determined by its salting-out capacity, which is associated with its Gibbs free energy of hydration (∆_hyd_*G*).

The SCN^−^ ion is weakly hydrated [48] and has a low salting-out capacity, which is confirmed by its ∆_hyd_*G* [49], so it is often considered a salting-in agent. The formation of an ABS has a different nature in the case of mixing KSCN and TBABr. The interaction between TBA^+^ and SCN^−^ is more favorable than the interaction of TBA^+^ with water and SCN^−^ with water. According to the phase diagram presented in Nakayama’s work [41], when adding a solid TBASCN to water, a two-phase liquid–liquid system is formed. Thus, it is likely that the new hydrophobic liquid phase released at mixing TBABr and KSCN aqueous solutions is a water-saturated TBASCN. The proposed procedure is very close to the method of in situ IL obtaining [50]. However, there are some differences. TBASCN salt is not formally IL, its melting point is 110 °C [51]. And, as shown above, when constructing the phase diagram, it is also not necessary to provide 1:1 stoichiometry, which is typical for in situ IL obtaining by mixing the solutions of cationic and anionic moiety suppliers. We found that the concentration of TBA^+^ and SCN^−^ in the upper phase obtained with the equimolar TBABr/KSCN ratio (*c*_t_ = 0.6 mol L^−1^) in the initial mixture is almost the same and amounts to 2.3 ± 0.2 and 2.34 ± 0.10 mol L^−1^, respectively. The concentration of bromide (0.076 ± 0.005 mol L^−1^) was thirty times less than those TBA^+^ and SCN^−^, with potassium below 1 × 10^−4^ mol L^−1^. In the lower aqueous phase, however, the Br^−^ and K^+^ concentrations were 0.27 ± 0.02 and 0.31 ± 0.02 mol L^−1^. This confirms that the upper phase consists of TBASCN saturated with water, produced by the exchange reaction: TBABr + KSCN = TBASCN + KBr. The measured water content in the upper phase was 0.77 ± 0.01 mole fraction (or 17.0 ± 0.6 wt%). The water content does not depend on the acidity in the range of pH 2.1–11.1. The high water content in the extracting phase is a characteristic feature of various ABSs, which is very valuable for solubilization and extraction of strongly hydrated highly polar/ionic compounds [31,34]. The upper phase is a transparent liquid with low viscosity (82.7 mPa·s, 25 °C) and a density (0.8660 g cm^−3^, 25 °C) lower than that of water. The relatively low viscosity of the upper phase is comparable to the viscosity of imidazolium ILs and of salt-salt ABSs, but significantly lower than the viscosity of ammonium-based ILs [34,52].

When aqueous solutions of TBABr and KSCN are mixed in a 1:2 ratio, the resulting upper phase is also predominantly TBASCN. This is confirmed by the ion concentrations in the upper phase (*c*_TBA_^+^ = 2.8 ± 0.2, *c*_SCN_^−^ = 2.70 ± 0.15, and *c*_Br_^−^ = 0.020 ± 0.005 mol L^−1^) and by elemental analysis (Tables S1 and S2). An excess of the thiocyanate ion reduces the TBA^+^ concentration in the lower phase, consistent with a common-ion effect.

The phase volume ratio depends on the total salt concentration (cₜ) and the molar ratio of the components. At an equimolar ratio of salts, increasing *cₜ* from 0.4 to 0.8 mol·L^−1^ reduces the lower-to-upper phase volume ratio from 44.7 to 9.0. Varying the molar ratio at constant *cₜ* also alters the upper phase volume, with KSCN having a greater effect due to the common-ion effect on TBASCN solubility (Table S3).

### 2.2. Distribution of Dyes in 1-Octanol–Water System

The extraction of dyes with an organic solvent is determined by the ability of extractants to form solvates, hydrate-solvate compounds, ionic associates, or hydrogen bonds. The structure of the dyes is equally critical: hydrophilic groups (–SO_3_^−^, –OH, –COOH) promote affinity for water, while aromatic moieties increase hydrophobicity. To correlate the partitioning behavior of dyes with the hydrophobicity parameter, their distribution between 1-octanol and water was studied.

**Figure 2:**
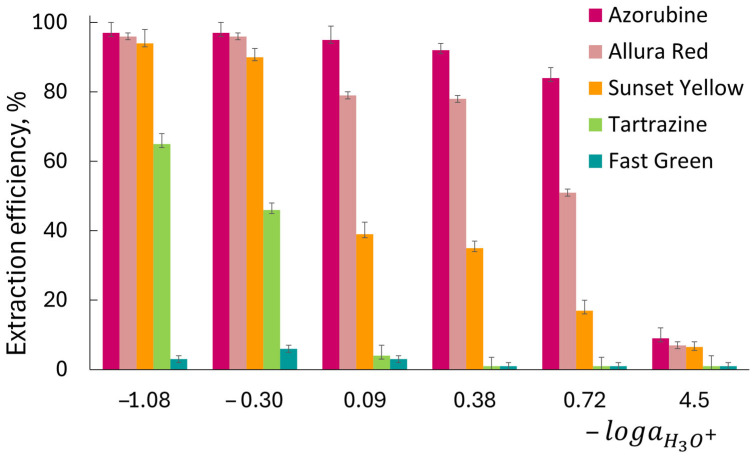
Distribution of dyes in 1-octanol–water system. (The H_3_O^+^ ions activity, $a_{H_{3}O^{+}},$ was calculated from the HCl concentrations using activity coefficients [44]). Dye concentration: Azorubine, Allura Red, Sunset Yellow, Tartrazine, 4.0 × 10^−5^ mol L^−1^; Fast Green, 8.0 × 10^−6^ mol L^−1^; *V*_aq.phase_ = *V*_octanol_ = 2.0 mL.

The distribution of dyes was investigated by varying the acidity of the aqueous solutions (Figure 2). For each HCl concentration used (5, 2, 1, 0.5, 0.25 and 5·× 10^−4^ mol L^−1^), the corresponding activity of H_3_O^+^ ions was calculated as $-loga_{H_{3}O^{+}}$, yielding values of −1.08, −0.3, 0.09, 0.38, 0.72, and 4.5, respectively. The activity coefficients for HCl at higher concentrations were taken from [44]. The studied food dyes contain at least two sulfonic and one hydroxyl group. Therefore, lowering the acidity leads to a higher proportion of the dyes in their anionic forms. Since the efficiency of their extraction with organic solvents decreases with the fraction of anions, the overall process becomes highly dependent on acidity. For all the anionic dyes studied, maximum extraction was achieved from 2 to 5 mol L^−1^ HCl solutions. Only Azorubine and Allura Red were quantitatively extracted into 1-octanol from 2 to 5 mol L^−1^ HCl. The extraction efficiency of Sunset Yellow was about 90% from 2 to 5 mol L^−1^ HCl, while Tartrazine was extracted only from a very acidic medium (2–5 mol L^−1^). The extraction efficiency of Fast Green was very low and did not exceed 7%. For all dyes studied, the equilibrium between the 1-octanol and aqueous phases was reached within 30 min (Figure S1). In accordance with the log *P* values obtained (from 5 mol L^−1^ HCl), the hydrophobicity of the dyes decreases in the following order: Azorubine (1.80 ± 0.02) > Allura Red (1.74 ± 0.03) > Sunset Yellow (1.50 ± 0.05) > Tartrazine (0.60 ± 0.04) > Fast Green (−1.15 ± 0.02). It is noteworthy that a similar tendency was also observed when studying the extraction of dyes with low-molecular-weight alcohols [53]. The log *D* values for the butanol-water system were 2.1 (Azorubine), 2.0 (Allura Red), 1.8 (Sunset Yellow), and 1.1 (Tartrazine). The decrease in solvent polarity with increasing length of the hydrocarbon chains explains the lower Log *P* values of the dyes.

### 2.3. Extraction of Dyes in TBABr–KSCN–H_2_O and TBABr–K_3_Cit–H_2_O

The preconcentration of dyes in ABS requires a selection of suitable phase-forming components due to the strong hydrophilic nature of the sulfonated dyes. Both TBABr-based systems are characterized by high water content in the upper phase and are promising for the extraction of hydrophilic dyes. In addition, the ionic nature of the extracting phase may provide the possibility of an ion exchange extraction mechanism or ion-pair extraction. Extraction from aqueous solutions was carried out for each dye separately.

#### 2.3.1. Extraction of Dyes in TBABr–KSCN–H_2_O

The extraction in TBABr–KSCN–H_2_O ABS was optimized by mixing aqueous solutions of TBABr and KSCN in a molar ratio of 1:1, *c_t_* = 0.4 mol·L^−1^. The volumes of the lower and upper phases were 3.80 ± 0.05 mL and 0.09 ± 0.01 mL, respectively.

**Figure 3:**
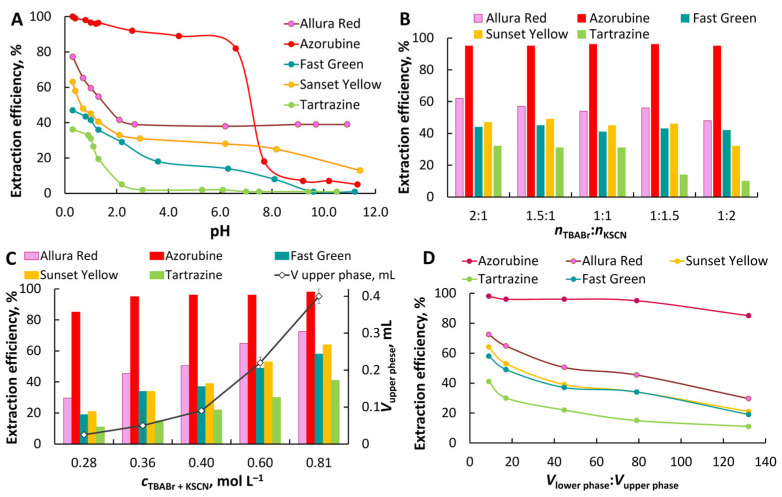
Effect of pH on dyes extraction in TBABr–KSCN–H_2_O ABS (**A**), *c*_TBABr_ = *c*_KSCN_ = 0.2 mol·L^−1^, *V*_lower phase_ = 3.8 mL and *V*_upper phase_ = 0.09 mL; effect of the molar ratio of TBABr:KSCN (**B**), 0.05 mol·L^−1^ HCl; effect of the total concentration of TBABr and KSCN (TBABr:KSCN = 1:1) and the volume of the upper phase (**C**), 0.05 mol·L^−1^ HCl; effect of the volume ratio (**D**), *c*_TBABr_ = *c*_KSCN_ = 0.2 mol·L^−1^, 0.05 mol·L^−1^ HCl. Dye concentration: Azorubine, Allura Red, Sunset Yellow, Tartrazine, 25 mg L^−1^; Fast Green, 8 mg L^−1^.

Effect of pH. The pH value determines the anionic state of the dye and affects its distribution between the organic and aqueous phases (Figure 3A). The maximum extraction efficiency for all dyes studied was observed from acidic solutions. The proportion of multiply charged dye anions increases with pH, leading to a gradual decrease in extraction. The TBABr–KSCN–H_2_O system appears to have a less pronounced ability to ion exchange compared to other TBABr-based ABSs formed via a salting-out effect. In this system, the extracting phase is water-saturated TBASCN. The exchange of a thiocyanate ion for a dye anion with the transfer of thiocyanate anions to the aqueous phase is less preferable compared to the possible exchange of bromide ions for dye anions. Extraction efficiency also depends on the nature of the dye. More hydrophobic dyes with a system of condensed benzene rings/naphthol fragments with alkyl substituents, for example, Azorubine, Allura Red, have higher extraction efficiency. Only Azorubine is quantitatively extracted from acidic solutions at pH ≤ 1.3 (≥0.05 M HCl), and at least 90% at pH 2.6–4.4. In contrast, dyes with many ionizing groups, like Fast Green and Tartrazine, were extracted less efficiently. The distribution coefficients (log *D*) for Azorubine, Allura Red, Fast Green, Sunset Yellow, and Tartrazine are 2.8 ± 0.2, 1.6 ± 0.1, 1.20 ± 0.05, 1.4 ± 0.1, and 1.10 ± 0.03 (from 0.1 mol L^−1^ HCl; *c*_TBABr_ = 0.2 mol L^−1^, *c*_KSCN_ = 0.2 mol L^−1^, *V*_lower_:*V*_upper_ = 44.7). The increase in dye extraction is in good agreement with the hydrophobicity of dyes estimated by the experimental Log *P*. For further work, the extraction of dyes was performed from 0.05 mol L^−1^ HCl.

Effect of TBABr:KSCN molar ratio, total phase-forming component concentration, and phase ratio. The effect of the molar ratio of TBABr: KSCN on the extraction of dyes is shown in Figure 3B. A slight increase in the extraction of some dyes was observed with a twofold excess of TBABr over KSCN compared to the equimolar ratio. During the extraction of anionic dyes, competition between thiocyanate ions and dye anions is possible for the formation of ion pairs with the tetrabutylammonium cation. An increase in the concentration of TBABr in this case should facilitate extraction. However, a slight improvement in extraction efficiency indicates the predominant role of hydrophobicity of extracted species.

The effect of *c*_t_ on the extraction efficiency was studied while maintaining an equimolar TBABr:KSCN ratio. An increase in *c*_t_ led to an increase in the volume of the upper phase (Figure 3C). However, quantitative extraction (≥97%) was achieved only for Azorubine in the range of 5.0 × 10^−2^–1 × 10^3^ mg L^−1^ from 0.05 mol L^−1^ HCl (*c*_TBABr_ = *c*_KSCN_ = 0.4 mol L^−1^).

Effect of extraction and centrifugation time. To evaluate the effect of phase contact time on the extraction efficiency of dyes the phase contact duration was varied from 30 s to 30 min. Maximum extraction efficiency for all dyes was achieved within 30 s (Figure S2). The increase in extraction time had no effect on the extraction efficiency. When TBABr and KSCN solutions are mixed, an exchange reaction occurs instantly. In fact, we were dealing with the dispersion of the extracting phase at the time of its formation. Increasing the contact area of the phases ensured fast and efficient mass transfer. To facilitate phase separation, the samples were centrifuged at 3000 rpm for 1–10 min. It was shown that 2 min of centrifugation was sufficient for complete separation. Without centrifugation, spontaneous phase separation occurred within approximately 10 min (Figure 1A).

Effect of the volume ratio on the extraction efficiency. To miniaturize the system, the phase ratio was adjusted by reducing the total concentration (*c*_t_) of the phase-forming components in the initial solution from 0.80 to 0.28 mol L^−1^ (*n*_TBABr_:*n*_KSCN_ = 1:1) at a constant volume of the sample. Preconcentration factors in the range from 132 to 9 were calculated as the volume ratio of the lower and upper phases. Figure 3D demonstrates the quantitative extraction of Azorubine at the volume of upper phase ≥ 50 μL. The volume ratio of the upper and lower phases is 1:80.

#### 2.3.2. Extraction of Dyes in TBABr–K_3_Cit–H_2_O

The preconcentration of dyes in the TBABr–K_3_Cit–H_2_O system can be ensured due to the high-volume ratio of the aqueous phase to the TBABr-enriched phase, which is achieved at low concentrations of TBABr and high concentration of a salting-out agent.

**Figure 4:**
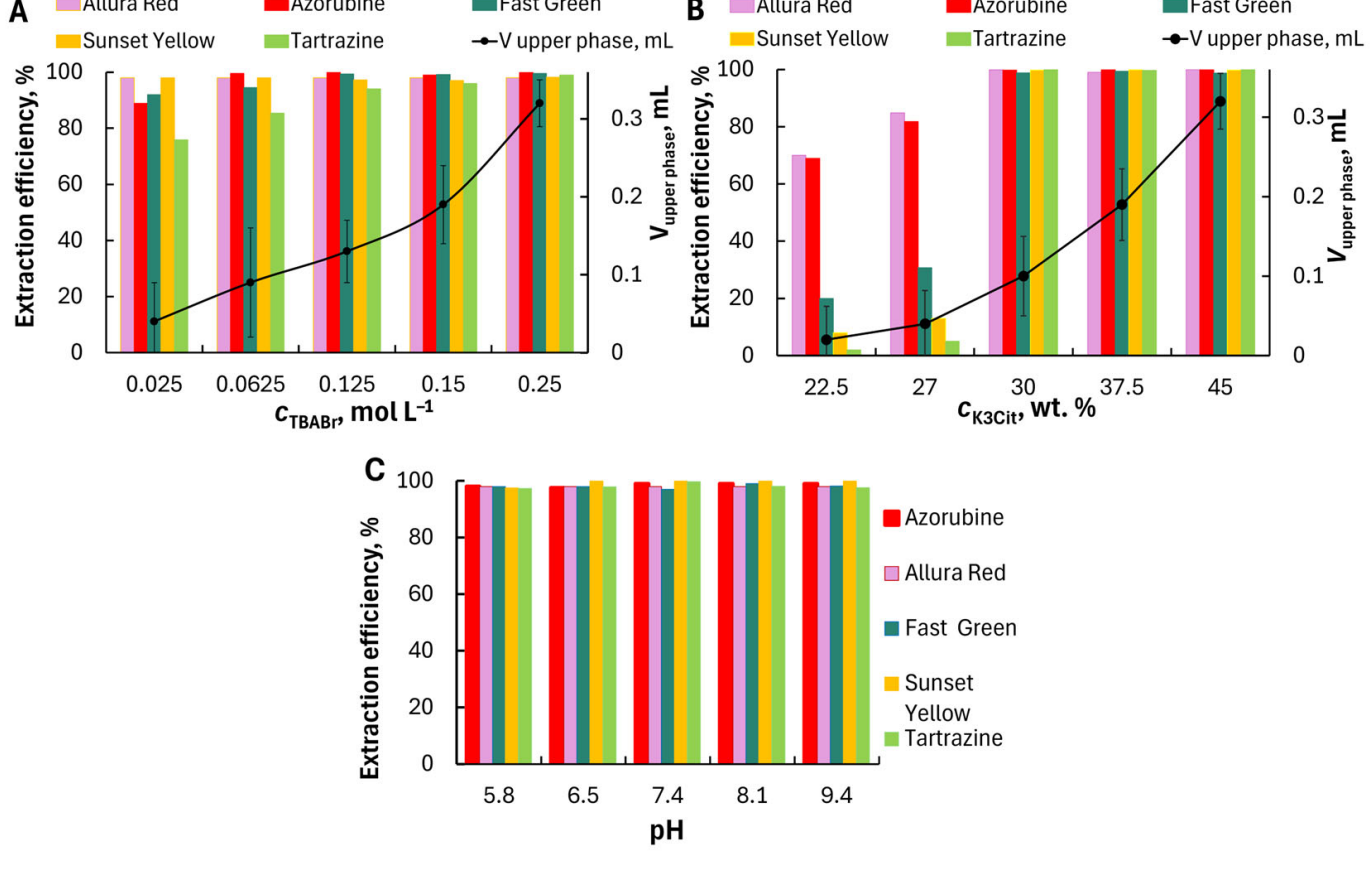
Effect of TBABr concentration on dyes extraction in TBABr–K_3_Cit–H_2_O ABS (**A**), *c*_K3Cit_ = 37.5 wt.%; effect of K_3_Cit concentration (**B**), *c*_TBABr_ = 0.15 mol·L^−1^; effect of pH (**C**), *c*_K3Cit_ = 30 wt.%, *c*_TBABr_ = 0.15 mol·L^−1^. Dye concentration: Azorubine, Allura Red, Sunset Yellow, Tartrazine, 25 mg L^−1^; Fast Green, 8 mg L^−1^.

Effect of phase-forming components, TBABr and K_3_Cit. To select conditions for the extraction preconcentration, we studied the effect of TBABr and K_3_Cit concentrations on the volume ratio of the lower and upper phases and on the extraction efficiency of dyes. The effect of the amount of TBABr in the range of 0.025–0.25 mol L^−1^ on the extraction efficiency of dyes was evaluated and shown in Figure 4A. The TBABr concentration significantly affected the volume of the separating upper phase and the phase volume ratio. The upper phase volume did not exceed 50 µL at 0.025 mol L^−1^ TBABr and 37.5 wt.% of potassium citrate. However, quantitative extraction was not achieved for all dyes. All the studied dyes were extracted quantitatively into TBABr–K_3_Cit–H_2_O system at 0.15 mol L^−1^ TBABr, pH 9.4; therefore, 0.15 mol L^−1^ TBABr was selected for subsequent experiments.

The effect of K_3_Cit concentration on the dye extraction was studied at 0.15 mol L^−1^ TBABr concentration. The formation of ABS occurs at the potassium citrate content ≥ 22.5 wt%. The extract volume increased from 0.03 to 0.32 mL with an increase in the concentration of K_3_Cit from 22.5 to 45 wt.%. All dyes were extracted quantitatively at the citrate concentration of 30 wt% (Figure 4B). For further studies, K_3_Cit concentration of 30 wt% and TBABr concentration of 0.15 mol L^−1^ were chosen to maintain the highest extraction efficiency.

Effect of pH. The effect of pH on dyes extraction was studied within the range of pH 5.8–9.4 (Figure 4C). Due to the high concentration of K_3_Cit, which creates an alkaline environment, it was not possible to investigate extraction over a wider pH range. All studied dyes were extracted quantitatively at pH 5.8–9.4 regardless of their ionic state in aqueous solution. The quantitative extraction of highly hydrated multiply charged anions is somewhat unusual. Unlike extraction with a conventional molecular solvent, the transfer of extracted species from the aqueous phase to the extracting phase of the ABS may not require a dehydration stage if both phases consist mainly of water. The measured water content was at least 35 wt.% (or χ ≥ 90%) for the TBABr-enriched phase. In addition, TBA^+^ cation is a phase-forming component and is present in high concentrations. It is well suited as a counter-ion for the formation of a hydrophobic pair/complex with a dye anion. It is most likely that when mixing solutions of TBABr and a dye, an ionic pair is formed because of electrostatic interaction. It is then extracted into the TBABr-enriched phase. The ionic nature of the organic phase provides the possibility of both the extraction of an ionic pair and the ion-exchange of bromide ions (components of the TBABr-rich phase) with the dye anions in the aqueous phase. The bromide ion can be easily exchanged for a more hydrophobic dye anion and transferred to the aqueous phase. The values of log *D* for all dyes were 3.4 ± 0.2, 3.3 ± 0.2, 3.50 ± 0.15, 3.4 ± 0.1 and 3.3 ± 0.1 for Azorubine, Allura Red, Sunset Yellow, Tartrazine and Fast Green, respectively (*V*_lower phase_:*V*_upper phase_ = 38.5:1).

Effect of phase contact time and centrifugation. The phase contact time was defined as the time between the moment of adding aqueous solutions of TBABr and K_3_Cit to the dyes solution and the start of the centrifugation. The extraction efficiency of all dyes was at least 98% after manual shaking for 15 s. The quantitative extraction achieved in less than 1 min is due to the fact that the formation of the system and the extraction of the dye occur simultaneously. When phase-forming components are added to an aqueous dye solution, droplets of a finely dispersed emulsion of the resulting organic phase are instantly formed. The dye anions are extracted into them immediately. The large surface area of the phase contact ensures rapid mass transfer. Phase separation occurs spontaneously within 5–10 min. Centrifugation can be used to accelerate the phase separation of samples. Centrifugation for 1 min (at 3000 rpm) is sufficient to achieve the most efficient and fastest separation.

### 2.4. Matrix Effect

The interference effects of several species on the extraction of dyes in TBABr -based ABSs were examined. The test samples of a solution containing 1 mg L^−1^ of Azorubine were subjected to the proposed procedures in the presence of different amounts of ascorbic, citric, glutamic acids, phenylalanine, saccharin and benzoate sodium salt, and others potentially interfering species. The tolerance concentration of the interfering substance was estimated as the highest amount of each compound that caused an error of less than ±5.0%. Possible interferences from various interfering substances were evaluated on the determination of Azorubine after extraction in TBABr–K_3_Cit–H_2_O and TBABr–KSCN–H_2_O separately. As can be seen in Table S4, the results demonstrated good recovery values (96–104%) which were achieved in the presence of most of the studied coexisting substances. This confirms the reliability of the proposed method for determination of selected dyes in real samples with complex matrices after preconcentration in tetrabutylammonium-based ABSs.

### 2.5. Quality Control and Quality Assurance

The analytical performance (calibration linearity, precision, limits of detection (LOD) and quantification (LOQ), preconcentration factor and other characteristics) was studied for preconcentration of Azorubine in the TBABr-KSCN-H_2_O ABS (*c*_TBABr_ = *c*_KSCN_ = 0.2 mol·L^−1^; *c*_HCl_ = 0.05 mol·L^−1^) and Azorubine, Allura Red, Sunset Yellow, Tartrazine, and Fast Green in the TBABr–K_3_Cit–H_2_O ABS (*c*_K3Cit_ = 30 wt.%, *c*_TBABr_ = 0.15 mol·L^−1^) under optimum experimental conditions.

The calibration plots were obtained over the range of 0.05–2.6 mg L^−1^ with eight concentration levels. Aqueous standard solutions of dyes were subjected to an entire procedure of the optimized microextraction in TBABr-based ABSs followed by the spectrophotometric determination. The LODs and LOQs were calculated as the three-fold and ten-fold standard deviations for 10 consecutive measurements of the blank solutions, respectively, to the calibration curves slopes [54]. The blank solutions were subjected to the entire method as well as the calibration ones. The recovery and intra-day and inter-day precision were determined at three concentration levels (0.08, 0.5 and 1.8 mg L^−1^ for each of the studied dyes) within one day (*n* = 3) and in several days (*n* = 5). The intra-day and inter-day repeatability was evaluated as relative standard deviation (RSD, %).

### 2.6. Method Validation

The proposed preconcentration procedure in TBABr-KSCN-H_2_O ABS was validated for Azorubine. The calibration plot was found to be linear in the range of 0.05–2.5 mg L^−1^ with a coefficient of determination (*R^2^*) equal to 0.9985. The LOD and LOQ were 0.015 and 0.05 mg L^−1^. The RSD values (%) for intra- and inter-day repeatability did not exceed 5.5% at three concentration levels for Azorubine (Table S5). The enhancement factor (EF) was defined as the ratio of the slope of the calibration curves after and before the developed extraction procedure and was 17.8. The preconcentration factor calculated as the phase volume ratio was 36.

The analytical performance data of the developed extraction procedure in TBABr–K_3_Cit–H_2_O ABS are summarized in Table 1. This method provided good linearity with good correlation coefficients (0.9976–0.9990). LODs were in the range of 0.006–0.02 mg L^−1^. The enhancement factors ranged from 14.3 to 19.3; and the preconcentration factor was 39. The RSD (%) values for intra- and inter-day repeatability did not exceed 5.4% at concentration 0.08, 0.5 and 1.80 mg L^−1^ for each of the studied dyes (Table 1). The recovery of samples ranged from 94% to 106%. This indicates the excellent reliability of the method.

### 2.7. Comparison of the Proposed Method with Other Reported Methods

According to our knowledge, this is the first report on the application of TBABr–KSCN–H_2_O ABS for the extraction and preconcentration. A comparison of the proposed preconcentration procedures of studied dyes in TBABr–based ABSs with recently reported data on the liquid–liquid microextraction (LLME) of synthetic food dyes is shown in Table 2. As can be seen, preconcentration in TBABr-based ABSs provides LODs that are close to other described methods of LLME of the studied dyes with the use of non-conventional “green” solvents. However, some extraction procedures need introduction of cooling stage in ice bath and/or a heating stage, which is time and energy consuming [5,24,25,26]. To increase the efficiency of extraction, dispersing solvents are often introduced, which are usually conventional organic solvents or DESs. In addition, the amount of a conventional solvent (usually hazardous substances) acting as a dispersing solvent may exceed the amount of solvent for extraction [5]. To prepare DESs and ILs, new different precursors and time are required [19]. Due to the high viscosity of some DESs and ILs, the introduction of dispersing solvents, increased temperature and ultrasound treatment are also often required [5,26,55]. Ultrasound treatment of the sample is often necessary to achieve quantitative extraction of dyes [55] and this can significantly affect the duration of the analysis [26].

The composition of extraction systems is often multicomponent due to the need to introduce additional substances that ensure the formation of a hydrophobic phase or an extractable compound [5,24]. All this complicates an extraction system and can lead to an unaccounted-for mutual influence when analyzing real objects. In the systems we propose, the phase-forming component is also the partner anion. A dispersant is not required, since the dispersion of extracting phase occurs spontaneously at the time of its formation. There are no heating or cooling stages. The centrifugation stage can be excluded, since phase separation occurs spontaneously and quickly, within 10 min. And perhaps most importantly, the phase-forming components are available. To prepare the extraction system, it is only necessary to mix two aqueous solutions of phase-forming components. There is no need to receive and store the extractant in advance. It can be obtained directly during the extraction process. Quantitative extraction of dyes is achieved in an extremely short period of time, less than one minute at room temperature.

### 2.8. Applications

To validate and prove the applicability of the method, real samples of drinks, jelly, and candy were tested by analyte addition recovery studies. Verification procedure was carried out by spiking known amounts of the target analyte to corresponding samples followed by quantitative analysis according to the proposed procedures. The results are given in Table 3. The recoveries ranged from 95% to 105% and RSDs were in the range of 1–6%, which confirms the accuracy and applicability of the method for the spectrophotometric quantification of dyes in food samples.

The results of Azorubine determination after extraction by the two ABSs were almost identical. The content of Azorubine in the analyzed food samples was estimated based on the results of two procedures. Taking into account the dilution of the initial sample, the dye content in Energy carbonated drink, Highly carbonated strawberry drink, Cotton candy flavored drink, Fruit jelly and Candy were 47 ± 2, 32 ± 2, 2.4 ± 0.2 mg L^−1^, 29.0 ± 1.5 and 18.3 ± 0.5 μg g^−1^ (*t* and *F* values, estimated at a 95% confidence level are given in Table S3). The content of Allura Red, Sunset Yellow and Tartrazine in beverages ranged from 2.8 to 73 mg L^−1^ (Table S6), which is in compliance with the relevant standards [10].

## 3. Materials and Methods

### 3.1. Instrumentation

A U-2900 double-beam UV-Visible spectrophotometer (Hitachi, Ibaraki Prefecture, Japan) equipped with a quartz microcuvette with a light path of 1 cm was used for absorbance measurements. The pH was measured using a pH-meter model pH-410 equipped with a combined glass microelectrode ELSK-13.7 (Aquilon, Moscow, Russia). A Hettich EBA-20 centrifuge (Tuttlingen, Germany) was employed to accelerate phase separation of the tetrabutylammonium-rich phase and aqueous phase. The weighing all chemicals was performed on a ViBRA HT (Shinko Denshi, Tokyo, Japan) analytical balance. The mixing of two-phase systems was performed with an ELMI S-3 orbital shaker (ELMI, Rīga, Latvia). A 870 KF Titrino plus titrator (Metrohm, Herisau, Switzerland) was used to measure the water content in the tetrabutylammonium-rich phase. A cone-and-plate CAP 2000+ viscometer (Brookfield, Chicago, IL, USA) was used to determine the viscosity of the sample. A Flash EA 1112 CHNS Analyzer (Thermo Electron Corp., Waltham, MA, USA) was used for elemental analysis. Dionex ICS 2100 Ion Chromatography system (Dionex part of Thermo Scientific, Sunnyvale, CA, USA) equipped with autosampler, an eluent electro-generation system, and a conductivity detector with ion suppressor was used for ion chromatographic analysis. Data acquisition and processing were controlled with Chromeleon 7 software (Dionex part of Thermo Scientific). An Agilent ICP-OES 720-ES inductively coupled plasma optical emission spectrometer operating in an axial viewing mode with an SPS3 autosampler and ICP Expert software 2.0.5 (Agilent Technologies, Santa Clara, CA, USA) was used for the detection of elements.

### 3.2. Chemicals and Samples

Tetra-*n*-butylammonium bromide, TBABr, (≥99%, Acros Organics, India), potassium thiocyanate, (≥98%, Prime Chemicals Group, shanghai, China), potassium citrate, K_3_Cit, (97%, Ruskhim, Moscow, Russia) were used as supplied. Food dyes Allura Red AC (≥80%), Azorubine (≥50%), Fast Green FCF (≥85%), Sunset Yellow FCF (90%) and Tartrazine (≥85%) were purchased from Sigma-Aldrich. Hydrochloric acids and sodium hydroxide (98%, Panreac, Darmstadt, Germany) were used for pH adjustments. 1-Octanol (99%, Panreac, Barcelona, Spain) was used for 1-octanol–water distribution coefficients determination. Ethanol (95%, Ekros, Saint Petersburg, Russia) was used to dilute of upper phase of ABS. All other reagents employed in this work were of analytical grade.

Stock solutions containing 2.0 × 10^−2^ mol L^−1^ of dyes were prepared by dissolving the required amount of dyes in distilled water. All working solutions were prepared daily by diluting the stock solutions in distilled water. 2.0 and 1.0 mol L^−1^ solutions of TBABr, KSCN and 60 wt% solutions of K_3_Cit were prepared by dissolving accurately weighed amounts of salts in distilled water.

Handmade filters obtained from Kimtech non-woven polypropylene polypropylene (NWPP) fiber (Kimtech Pure W4, Kimberly-Clark, TX, USA) were used for filtration.

### 3.3. Preparation of Aqueous Biphasic Systems

#### 3.3.1. Formation and Properties of TBABr–KSCN–H_2_O System

For the phase diagrams construction, the aqueous solutions of 0.5, 1.0, 1.5, and 2.0 mol L^−1^ TBABr and KSCN were initially prepared by dissolving weighed amounts of the salts (±10^−4^ g) in distilled water. The binodal curve was determined at room temperature 22 ± 2 °C by turbidimetric titration [58]. Two series of samples were prepared, each containing only one phase-forming component, TBABr or KSCN, at variable concentration. The samples were titrated with the solution of the other phase-forming component under constant stirring until the turbidity of the solution was observed. The turbidity is an indication that the system has become heterogeneous, as a result of a new phase formation. The concentrations of TBABr and KSCN at the endpoints of titration correspond to the points on a binodal curve (Table S7).

To determine the concentration of ions (Br^−^, SCN^−^, TBA^+^, K^+^) in the upper and lower phases, ABSs were obtained by mixing 1.0 mol L^−1^ aqueous solutions of TBABr and KSCN. The total concentration of phase-forming components (*c*_t_ = *c*_TBABr_ + *c*_KSCN_) was constant and equal to 0.600 mol L^−1^. The molar ratio of TBABr:KSCN were 2:1, 1:1, and 1:2. The samples were mixed by simple manual stirring and left to achieve equilibrium at room temperature for at least 48 h (22 ± 2 °C). Initially, 50 or 100 µL of the upper phases were diluted 20 times with 25% ethanol (*v*/*v*), and 500 µL of the lower phases were diluted 5 times with distilled water. Before chromatographic determination, if necessary, the samples were diluted with distilled water. The chromatographic procedure for determination of Br^−^ and SCN^−^ with utilization of stationary phase described in [59] is given in detail in Supplementary Materials. The concentration of TBA^+^ in upper and lower phases was determined by UV–Vis spectroscopy after extraction into chloroform with picric acid [60]. The concentration of potassium was determined by ICP-OES.

#### 3.3.2. Formation of TBABr–K_3_Cit–H_2_O System

The phase diagram was constructed using the turbidimetric titration at 22 ± 2 °C [58]. To prepare the TBABr–H_2_O–K_3_Cit aqueous biphasic system and determine the liquid–liquid phase separation boundaries, the stock solutions of TBABr (1.0 mol L^−1^) and K_3_Cit (2.57 mol L^−1^ or 60.7 wt%) were prepared by dissolving accurately weighed amounts of salts (±10^−4^ g) in distilled water. Two-phase systems were obtained by mixing the stock solutions of TBABr and K_3_Cit in the volume ratios of *V*_TBABr_:*V*_K3Cit_ from 3:1 to 1:99 with a total volume of 10.0 mL. The samples were titrated with distilled water, drop-wise, under constant stirring with a magnetic stirrer until the system just turns clear, i.e., one phase is formed. The single-phase point of the system (transparent solution) was visually detected. The volume of water spent on the titration was measured and the concentrations of TBABr and K_3_Cit at the end point of the titration were calculated. The calculated concentrations correspond to the points on the binodal curve (Table S8).

### 3.4. Extraction Procedure

Extraction was performed at room temperature (21 ± 3 °C). A 0.4 mL aliquot of dye solution was placed into a glass tube. Then, the phase-forming components of TBABr/KSCN or TBABr/K_3_Cit ABSs were added.

TBABr–KSCN–H_2_O system: 0.8 mL of 1.0 mol L^−1^ KSCN, 0.8 mL of 1.0 mol L^−1^ TBABr and 2.0 mL of distilled water were added to the dye aliquot.

TBABr–K_3_Cit–H_2_O system: 0.5 mL of 1.0 mol L^−1^ TBABr, 2.5 mL of 60 wt.% K_3_Cit were added to the dye aliquot. The total volume was completed to 4.0 mL with distilled water. If necessary, 5.0 and 5.0 mol L^−1^ HCl were added to the aqueous phase was to adjust the pH.

The mixtures were stirred manually for 30 s, after which the samples were centrifuged for 2 min at 3000 rpm. Phase separation was achieved by passing the sample through a filter from NWPP. The TBABr-enriched phase was completely captured by the filter, and the aqueous phase passed freely through it. After separation, the pH of the aqueous phase was measured, and the residual content of dyes was determined by UV–Vis spectroscopy. The extraction efficiency (*E*, %) and the distribution ratio (*D*) of dyes were calculated as:

$$E\left(\%\right)=100\cdot (1-\frac{c_{aq}V_{aq}}{c_{aq}^{0}V_{aq}^{0}})$$

$$D=\frac{c_{aq}^{0}-c_{aq}}{c_{aq}}\cdot \frac{V_{aq}}{V_{o}}$$

where $c_{aq}^{0}$ and $c_{aq}$ are the initial and final (equilibrium) concentrations (mol L*^−^*^1^) of a dye in the aqueous phase, respectively; $V_{o}$ is the volume of organic (upper) phase; $V_{aq}^{0}$ and $V_{aq}$ are the initial and final volumes (mL) of the aqueous phase, respectively. All experiments were repeated at least three times, and the standard deviation of the results was within 5% (unless otherwise stated as error bars).

### 3.5. Preparation of Real Samples

Commercial food samples such as soft drinks, candies, fruit jelly containing Azorubine, Allura Red, Sunset Yellow, Tartrazine and Fast Green, marked on their packages, were purchased in a local supermarket (Moscow, Russia). The beverage samples were diluted to bring their concentration to an acceptable level. The pre-treatment processes performed for real samples are given in detail in Table S6. 1.500 g of fruit jelly and candies were individually dissolved in 50.0 mL of distilled water at moderate heat (up to 70 °C). The resulting mixture was mixed and filtered through NWPP.

Extraction in ABS TBABr–K_3_Cit–H_2_O. An 8.0 mL aliquot of the real sample was placed in a 15 mL polypropylene centrifuge tube, and 2.0 mL of 1.0 mol L^−1^ TBABr and 4.90 g of K_3_Cit were added.

Extraction in ABS TBABr–KSCN–H_2_O. An 8.0 mL aliquot of the real sample was placed in a 15 mL polypropylene centrifuge tube, and 1.2 mL of 2.0 mol L^−1^ KSCN, 1.2 mL of 2.0 mol L^−1^ TBABr and 100 μL of 5 mol L^−1^ HCl were added.

The mixtures were stirred manually for 30 s, after which the samples were centrifuged for 2 min at 3000 rpm. A 200 µL of the extract was pipetted into a clean tube, and then 50 µL of distilled water (for TBABr/K_3_Cit) or 80 µL (for TBABr/KSCN) of ethanol was added. The content of dyes was determined by UV–Vis spectroscopy. The same procedure was carried out in cases of blank solutions and calibration samples. The blank solution contained all the phase-forming components with the exception of the dye and was fully processed throughout the method. The blank solution was prepared similarly to the calibration samples. All phase-forming components were added to 8.0 mL of distilled water. After shaking, centrifugation, and phase separation, 50 µL of distilled water (for the TBABr/K3Cit) or 80 µL of ethanol (for the TBABr/KSCN) was added to 200 µL of the organic phase, and the absorption was measured.

### 3.6. Measurement of Octanol-Water Partition Coefficient

The octanol−water partition coefficients were measured using the traditional shake-flask method, [42,61] which is characterized by ease of operation, low cost and accuracy.

1-Octanol and aqueous solutions of 0.25, 0.5, 1.0, 2.0, 5.0 mol L^−1^ HCl, and 0.1 mol L^−1^ NaOH were pre-saturated by stirring for 12 h in equal volumes of each other. After that, the mixtures were poured into a separation funnel and left overnight. Subsequently, these two layers were separated and stored for further studies. An 100 μL aliquot of an aqueous solution containing 8.0 × 10^−4^ mol L^−1^ dye (1.6 × 10^−4^ mol L^−1^ for Fast Green FCF) was placed in a 10 mL glass tube. Then, 2.0 mL of 1-octanol saturated with aqueous solutions of either HCl or NaOH, and 1.9 mL of the corresponding HCl or NaOH solution saturated with 1-octanol were added successively. The mixture was stirred for 2 h, then poured into a separation funnel and left to stand for 2 h. After this, the phases were separated and the absorption spectra of the aqueous and organic phases were recorded. Dye concentrations in the aqueous phase were determined by UV-Vis spectroscopy. Standards with known dye concentrations of different acidity were prepared and used to perform calibrations of absorbance vs. concentration. Analysis by paper chromatography (3MM paper with isobutanol-ethanol-water, 3:1:1 solvent [62]) showed a single colored component in each of the dyes used. The concentration of dye in 1-octanol was calculated from the mass balance. Each experiment was performed three times. The octanol–water partition coefficient (log *P*) was calculated as follows:

$$\log P=\;log\frac{c_{o}}{c_{aq}},$$

where $c_{o}$ is the concentration of dye in the octanol phase and $c_{aq}$ is its concentration in the aqueous phase when the system is at equilibrium.

## 4. Conclusions

For the first time, the novel ABS based on TBABr and KSCN was applied in the extraction/preconcentration of dyes followed by spectrophotometric determination. A distinctive feature of the TBABr–KSCN–H_2_O system is a wide two-phase region in which the phase-forming components, TBABr and KSCN, are present in comparable concentrations, unlike ABSs formed due to the salting-out effect. The extraction efficiency of dyes in the TBASCN-enriched phase depends on their hydrophobicity. The increase in dye extraction is in good agreement with their hydrophobicity, as indicated by the experimental values of log *P*. All dyes studied are quantitatively extracted into TBABr–K_3_Cit–H_2_O ABS formed by salting-out effect regardless of their hydrophobicity. This system exhibits a more pronounced ability to ion exchange compared to TBABr–KSCN–H_2_O ABS. The exchange of the bromide ion for the dye anion with its transfer to the aqueous phase is preferable compared to the possible exchange of thiocyanate ions for dye anions. For both ABSs, optimal working conditions were established to ensure quantitative extraction and pre-concentration. The practical applicability of ABSs was successfully demonstrated through the extraction of dyes from food samples.

The developed ABS-based preconcentration methods present sustainable and practical alternatives to conventional liquid–liquid extraction. The key advantage is the replacement of toxic, volatile organic solvents with benign aqueous phases formed by simple salts. These systems contain no volatile components, exhibit high water content in both phases, and are inherently non-flammable and of low toxicity, ensuring a low environmental impact. A major operational benefit of the studied ABSs is the broad concentration range over which two-phase formation occurs. This tunability allows precise control of the phase volume ratio, facilitating the isolation of a small extract volume from a large sample and thereby achieving high preconcentration factors. The resulting extracts have low viscosity and are water-miscible, enabling direct analysis (e.g., by spectrophotometry or chromatography) without the need for solvent exchange or the use of additional organic solvents. Furthermore, these methods are designed for microscale operation, which minimizes reagent consumption and waste generation. All phase-forming components are commercially available, inexpensive, and straightforward to handle.

In summary, the proposed TBABr-based salt–salt ABSs provide efficient, tunable, and environmentally benign platforms for extraction. They are particularly promising for the preconcentration and analysis of hydrophilic compounds, such as synthetic dyes.

## Figures and Tables

**Table 1:** Analytical characteristics for the spectrophotometric determination of dyes after extraction in TBABr–K_3_Cit–H_2_O ABS.

Parameter	Azorubine	Allura Red	Fast Green	Sunset Yellow	Tartrazine
Calibration curve equation, *A* = *ac* + *b*, mg L^−1^	A = 0.9991*c* + 0.069	A = 0.8961*c* + 0.015	A = 2.399*c* + 0.047	A = 0.8163*c* + 0.007	A = 0.7585*c* + 0.053
*R* ^2^	0.9977	0.9977	0.9990	0.9987	0.9976
*λ*, nm	528	516	611	485	430
Linearity range, mg L^−1^	0.05–2.60	0.05–2.50	0.02–0.75	0.06–1.90	0.06–2.30
LOD, mg L^−1^	0.01	0.02	0.006	0.02	0.02
LOQ, mg L^−1^	0.05	0.05	0.02	0.06	0.06
Intra-day precision (RSD, %, *n* = 3)					
0.08 mg L^−1^	2.5	2.4	1.5	2.6	1.5
0.5 mg L^−1^	1.6	1.5	1.7	0.8	0.8
1.8 mg L^−1^	1.1	1.1	1.5 ^1^	2.3	2.3
Inter-day precision (RSD, %, *n* = 5)					
0.08 mg L^−1^	5.0	5.4	3.5	3.5	4.0
0.5 mg L^−1^	2.0	2.8	2.3	2.0	2.2
1.8 mg L^−1^	2.8	3.5	1.5 ^1^	3.8	2.9
Enhancement factor	19.3	16.6	16	14.3	17
Added concentration, mg L^−1^	0.08	0.08	0.08	0.08	0.08
Found concentration ^2^, mg L^−1^	0.080 ± 0.005	0.085 ± 0.005	0.080 ± 0.003	0.075 ± 0.005	0.083± 0.003
Recovery, %	100 ± 6	106 ± 6	100 ± 4	94 ± 6	104 ± 4
Added concentration, mg L^−1^	0.5	0.5	0.5	0.5	0.5
Found concentration ^2^, mg L^−1^	0.50 ± 0.02	0.52 ± 0.02	0.48 ± 0.02	0.50 ± 0.01	0.51 ± 0.01
Recovery, %	100 ± 4	104 ± 4	96 ± 4	100 ± 2	102 ± 2
Added concentration, mg L^−1^	1.80	1.80	0.75	1.80	1.80
Found concentration ^2^, mg L^−1^	1.80 ± 0.05	1.85 ± 0.05	0.78 ± 0.03	1.75 ± 0.10	1.72 ± 0.10
Recovery, %	100 ± 3	103 ± 3	104 ± 4	97 ± 6	96 ± 6

^1^ RSD for spiked level 0.75 0 mg L^−1^, ^2^ Mean and confidence interval (*P* = 0.95, *n* = 3).

**Table 2:** Comparison of some analytical characteristics of proposed procedures with other extraction preconcentration methods.

Sample	Dye	Extraction Procedure ^1^	Extraction System ^2^	Analytical Method ^3^	Linearity Range, μg L^−1^	LOD ^4^, μg L^−1^	Ref.
Candy, soft drinks and jellies	Allura Red Tartrazine, Fast Green	IP-SA-DLLME-SFOD	1-Undecanol/CTAB	UV–Vis	15–1500 35–2000 3–1200	4 10 0.8	[5]
Candy, energy drink	Allura Red	VA-SS-LPME	DES (TBABr/ 1-Nonanol)	UV–Vis	172–8000	51	[22]
Energy drink, jelly, candy	Allura Red	DLLME-SFO	DES (Choline chloride/Citric Acid)	UV–Vis	100–5000	30	[24]
Lipstick, pastille	Fast Green	LLME-DES	DES (TBABr/ Decanoic acid)	HPTLC	100–1200	80	[25]
Beverages	Sunset Yellow	IL-ABS	Choline amino acid/K_3_PO_4_	HPLC-UV	50–40,000	21	[19]
Candied fruit, cotton candy and confectionery sprinkles	Sunset Yellow Azorubine	LLME-QH-DES	DES (TBABr/ Hexanoic acid)	HPLC	1–100 1–100	0.1 0.1	[26]
Candy, energy drink, syrup, juice powder	Tartrazine, Sunset Yellow, Allura Red	VA-IPS-LLME	TBAI and 1-pentanol	UV–Vis	230–15,000 160–20,000 190–12,000	67 46 54	[30]
Candy, carbonated drink	Tartrazine, Sunset Yellow		BMImBr/K_2_HPO_4_	HPLC	0.61–2 0.58–2	0.05 0.05	[38]
Syrup	Sunset Yellow	UA-DLLME-DES	DES (TBABr/Decanoic acid)	UV–Vis	–	0.05	[55]
Solid juice, solid jelly	Sunset Yellow	SALLE	Acetonitrile/(NH_4_)_2_SO_4_	UV–Vis	400–1500	70	[56]
Beverage, Easter set	Allura Red Azorubine	THABr—ABS	THABr	UV–Vis	50–4950 50–2000	4 6	[57]
Soft drinks, jellies, candies	Allura red Azorubine Fast Green Sunset Yellow Tartrazine Azorubine	TBABr-K_3_Cit-ABS TBABr-KSCN-ABS	TBABr/K_3_Cit TBABr/KSCN	UV–Vis	50–2500 50–2600 20–750 50–1900 50–2300 50–2500	20 10 6 20 20 20	This Work

^1^ IL-ABS—Ionic liquid-based aqueous biphasic systems; UA-DLLME-DES—Ultrasonic assisted dispersive liquid–liquid microextraction based on deep eutectic solvent; SALLE—Salting-out assisted liquid–liquid extraction; DLLME-SFO—Dispersive liquid–liquid microextraction based on the solidification of a floating organic drop; VA-SS-LPME—Vortex assisted sequential-simultaneous liquid phase microextraction; IP-SA-DLLME-SFOD—Ion-pair-based surfactant-assisted dispersive liquid–liquid microextraction based on the solidification of a floating organic drop; LLME-DES—Liquid–liquid microextraction with deep eutectic solvent; LLME-QH-DES—Liquid–liquid microextraction with quasi-hydrophobic deep eutectic solvent; VA-IPS-LLME–Vortex-assisted ion-pair solvent-based liquid–liquid microextraction; THABr-ABS–Tetrahexylammonium bromide-based aqueous biphasic system; TBABr-K3Cit-ABS—Tetrabutylammonium bromide-based aqueous biphasic system; TBABr-KSCN-ABS—Tetrabutylammonium thiocyanate-based aqueous biphasic system. ^2^ TBABr—tetrabutylammonium bromide; CTAB—cetyltrimethylammonium bromide, TBAI–tetra-*n*-butyl ammonium iodide; BMImBr—1-butyl-3-methylimidazolium bromide; THABr—tetrahexyl-ammonium bromide; K_3_Cit–potassium citrate. ^3^ HPLC-UV—High-performance liquid chromatography with spectrophotometric detection; UV–Vis–Ultraviolet visible spectrometry; HPTLC—High-performance thin layer chromatography. ^4^ Authors’ data from the original papers were adjusted by rounding off the numbers to show the appropriate number of significant digits.

**Table 3:** The results of extraction-photometric determination of dyes in food samples (*n* = 3, *p* = 0.95).

Added, mg L^−1^	Found *, mg L^−1^	RSD_,_ %	Recovery, %	Added, mg L^−1^	Found *, mg L^−1^	RSD, %	Recovery, %
TBABr–K_3_Cit–H_2_O				TBABr–KSCN–H_2_O			
Azorubine							
	Energy carbonated drink						
0	0.47 ± 0.04	3	–	0	0.48 ± 0.05	4	–
0.31	0.80 ± 0.06	3	104	0.31	0.79 ± 0.05	3	100
0.62	1.10 ± 0.10	4	102	0.62	1.08 ± 0.04	2	96
	Highly carbonated strawberry drink						
0	0.33 ± 0.02	2	–	0	0.31 ± 0.03	3	–
0.31	0.65 ± 0.04	3	103	0.31	0.62 ± 0.04	3	100
0.62	0.95 ± 0.05	4	100	0.62	0.92 ± 0.04	2	97
	Cotton candy flavored drink						
0	0.25 ± 0.02	3	–	0	0.23 ± 0.01	2	–
0.31	0.55 ± 0.03	3	96	0.31	0.53 ± 0.02	2	96
0.62	0.88 ± 0.02	1	104	0.62	0.84 ± 0.04	2	96
	Mini fruit jelly “Cherry flavor” **						
0	28 ± 2	3	–	0	30 ± 3	4	–
10	38 ± 2	3	100	10	39 ± 2	2	97
20	49 ± 3	2	104	20	51 ± 3	2	103
	Candy **						
0	18 ± 1	2	–	0	19 ± 1	1	–
10	29 ± 2	3	105	10	29 ± 1	2	103
20	39 ± 4	4	105	20	38 ± 2	2	97
TBABr–K_3_Cit–H_2_O							
Allura Red							
	Strawberry-cream carbonated drink			Strawberry-flavored carbonated soft drink			
0	0.18 ± 0.02	4	–	0	0.73 ± 0.04	2	–
0.30	0.48 ± 0.02	2	100	0.30	1.05 ± 0.05	2	103
0.60	0.78 ± 0.04	2	100	0.60	1.30 ± 0.10	3	96
	Vitamin strawberry-vanilla drink			Mini fruit jelly “Peach flavor” **			
0	1.05 ± 0.05	2	–	0	5.5 ± 0.5	4	–
0.5	1.60 ± 0.05	1	105	5.5	11.2 ± 0.2	2	104
1.5	2.50 ± 0.10	2	95	11.0	16.3 ± 0.3	1	96
Sunset Yellow							
	Vitamin mango and kiwi drink			Orange-flavored carbonated drink			
0	0.28 ± 0.02	3	–	0	0.36 ± 0.02	2	–
0.32	0.61 ± 0.03	2	104	0.32	0.70 ± 0.04	2	105
0.64	0.92 ± 0.06	3	100	0.64	1.00 ± 0.08	3	100
Sunset Yellow				Fast Green			
	Mini fruit jelly “Passion flavor” **			Mouthwash “Listerine”			
0	12 ± 1	2	–	0	0.15 ± 0.01	3	–
10	21 ± 2	4	96	0.10	0.25 ± 0.02	3	100
20	32 ± 3	4	104	0.20	0.35 ± 0.04	5	100
Tartrazine							
	Carbonated drink with cream flavor			Carbonated drink “Mountain Dew”			
0	0.28 ± 0.04	6	–	0	0.63 ± 0.06	4	–
0.25	0.54 ± 0.04	3	104	0.53	1.17 ± 0.03	2	102
0.53	0.80 ± 0.05	2	96	1.06	1.68 ± 0.08	2	98

* Mean and confidence interval (*p* = 0.95, *n* = 3). ** The dye content is expressed in μg g^−1^.

## Data Availability

The original contributions presented in the study are included in the article/Supplementary Materials, further inquiries can be directed to the corresponding author.

## Supplementary Materials

The following supporting information can be downloaded at: https://www.mdpi.com/article/10.3390/molecules30244769/s1, Figure S1: Effect of phase contact time on dyes extraction efficiency in 1-octanol–H_2_O system; Figure S2: Effect of phase contact time on dyes extraction efficiency in TBABr–KSCN–H_2_O ABS; Table S1: Determination of bromide, thiocyanate, potassium and tetrabutylammonium in the upper and lower phases of TBABr–KSCN–H_2_O ABS; Table S2: Data of the elemental analysis of upper phase of TBABr–KSCN–H_2_O ABS; Table S3: The phase volume ratio for TBAB–KSCN–H_2_O ABS; Table S4: Effect of interfering some chemicals on the recovery of Azorubine; Table S5: Analytical characteristics for the spectrophotometric determination of Azorubine after extraction in TBABr–KSCN–H_2_O ABS; Table S6: The pre-treatment processes performed for real samples; Table S7: Data for plotting the TBABr–KSCN–H_2_O diagram; Table S8: Data for plotting the TBABr–K_3_Cit–H_2_O diagram; Chromatographic procedure for determination of Br^−^ and SCN^−^ [59].
