# Supplementary material for: Aqueous Biphasic Systems Based on Tetrabutylammonium Bromide for Extraction and Determination of Azorubine, Allura Red, Sunset Yellow, Tartrazine and Fast Green in Food Samples
Source: Molecules. 2025 Dec 13;30(24):4769. doi: 10.3390/molecules30244769 (PMC12735457; doi:10.3390/molecules30244769)
Supplement: Supplementary file 1 [file molecules-30-04769-s001.zip › molecules-3988771-supplementary.pdf]

Supplementary materials

Aqueous biphasic systems based on tetrabutylammonium bromide for extraction and determination of Azorubine, Allura Red, Sunset Yellow, Tartrazine and Fast Green in food samples

Svetlana V. Smirnova [1,*], Anastasia V. Gorbovskaia [1], Yulia S. Vershinina [1,2], Vladimir V. Apyari [1], and Mikhail A. Proskurnin [1]

[1] Chemistry Department, Lomonosov Moscow State University, Leninskie Gory 1, Moscow 119991, Russia

[2] Federal State Budgetary Institution of Science Institute of African Studies, Russian Academy of Sciences, St. Spiridonovka, 30/1, Moscow 123001, Russia

* Corresponding author at: Chemistry Department, Lomonosov Moscow State University, Leninskie Gory 1, Moscow 119991, Russia

E-mail address: sv_v_smirnova@mail.ru

Table of contents

Table S1 Determination of of bromide, thiocyanate, potassium and tetrabutylammonium in the upper and lower phases of TBABr–KSCN–H_2O ABS

Table S2 Data of the elemental analysis of upper phase of TBABr–KSCN–H_2O ABS

Table S3 The phase volume ratio for TBAB–KSCN–H_2O ABS.

Table S4 Effect of interfering some chemicals on the recovery of Azorubine

Table S5 Analytical characteristics for the spectrophotometric determination of Azorubine after extraction in TBABr–KSCN–H_2O ABS.

Table S6 The pre-treatment processes performed for real samples.

Table S7 Data for plotting the TBABr–KSCN–H_2O diagram.

Table S8 Data for plotting the TBABr–K_3Cit–H_2O diagram.

Fig. S1 Effect of phase contact time on dyes extraction efficiency in 1-octanol–H_2O system.

Fig. S2 Effect of phase contact time on dyes extraction efficiency in TBABr–KSCN–H_2O ABS.

Chromatographic procedure for determination of Br^- and SCN^-

References

Table S1 Determination of bromide, thiocyanate, potassium and tetrabutylammonium in the upper and lower phases of TBABr–KSCN–H_2O ABS

Stock solutions, mol L^{-1}		Molar ratio	Concentration of phase-forming components in upper phase, mol L^{-1}				Concentration of phase-forming components in lower phase, mol L^{-1}			
TBABr	KSCN	TBABr/ KSCN	SCN^-	TBA^+	Br^-	K^+,	SCN^-	TBA^+	Br^-	K^+
0.2	0.4	0.5	2.70 ± 0.15	2.8 ± 0.2	0.020 ± 0.005	$< 1\cdot10^{-4}$	0.26 ± 0.05	0.077±0.003	0.22 ± 0.02	0.38 ± 0.01
0.4	0.2	2	2.65 ± 0.15	2.8 ± 0.2	0.21 ± 0.01	$< 1\cdot10^{-4}$	0.08 ± 0.01	0.24 ± 0.02	0.43 ± 0.03	0.22 ± 0.01
0.3	0.3	1	2.34 ± 0.10	2.3 ± 0.2	0.076 ± 0.005	$< 1\cdot10^{-4}$	0.13 ± 0.05	0.105±0.005	0.27 ± 0.02	0.31 ± 0.02

Table S2 Data of the elemental analysis of upper phase of TBABr–KSCN–H_2O ABS*

	Weight percentages (wt.%)				C/N	C/S	N/S
	C	H	N	S			
Calculated contents (TBASCN)	58.07	11.17	7.97	9.12	7.29	6.37	0.87
Experimental data (TBABr:KSCN = 1:1)	58.13	10.4	8.2	9.74	7.09	5.97	0.84
Experimental data (TBABr:KSCN = 1:2)	58.4	10.27	8.3	10.3	7.04	5.67	0.81

* The ABS was obtained by mixing aqueous solutions of TBABr and KSCN at the molar ratio of TBABr:KSCN equal 1:1 and 1:2 (c_t = 0.6 mol L^{-1})

Table S3 The phase volume ratio for TBAB–KSCN–H_2O ABS

TBABr:KSCN molar ratio		Total concentration $c_{TBABr} + c_{KSCN}$, mol L^{-1}		
		0.4	0.6	0.8
1:1	$V_{upper\ phase}$, mL	0.09 ± 0.01	0.22 ± 0.05	0.40 ± 0.05
	$V_{lower} : V_{upper}$	44.7	17.1	9.0
2:1	$V_{upper\ phase}$, mL	0.06 ± 0.05	0.12 ± 0.05	0.25 ± 0.05
	$V_{lower} : V_{upper}$	62	29	13
1:2	$V_{upper\ phase}$, mL	0.07 ± 0.05	0.17 ± 0.05	0.35 ± 0.05
	$V_{lower} : V_{upper}$	53	22	11

Table S4 Effect of some interfering chemicals on the recovery of Azorubine[a]

Interfering species	TBABr-KSCN-H_2O		TBABr-K_3Cit-H_2O	
	Ratio to Azorubine	Recovery[b] (%)	Ratio to Azorubine	Recovery[b] (%)
Ascorbic acid	1000	100 ± 2	1000	102 ± 5
Citric acid	1000	98 ± 2	1000	98 ± 3
Glutamic acid	500	102 ± 3	500	99 ± 3
Phenylalanine	500	97± 4	500	103 ± 4
Sugar	1000	96 ± 5	1000	100 ± 2
Saccharin sodium salt	1000	102 ± 4	1000	97 ± 4
Sodium benzoate	500	101 ± 3	500	97 ± 3
Fe^{3+}	1	102 ± 2	100	103 ± 2
Na^{+}, SO_4^{2-}	1000	99 ± 4	1000	97 ± 4
K^{+}, Cl^{-}	1000	102 ± 4	1000	104 ± 4

[a] 1.0 mg L^{-1} Azorubine

[b] recovery experiments were performed in triplicates

Table S5 Analytical characteristics for the spectrophotometric determination of Azorubine after extraction in TBABr–KSCN–H_2O ABS

Intra-day precision	Added concentration, mg L^{-1}	Found concentration[a], mg L^{-1}	RSD, % (n = 3)	Recovery, %
0.08 mg L^{-1}	0.08	0.080 ± 0.006	2.7	100
0.5 mg L^{-1}	0.5	0.51 ± 0.01	1.1	102
1.8 mg L^{-1}	1.8	1.75 ± 0.06	1.4	97
Inter-day precision			RSD, % (n = 5)	
0.08 mg L^{-1}	0.08	0.075 ± 0.005	5.5	94
0.5 mg L^{-1}	0.5	0.51 ± 0.01	1.9	102
1.8 mg L^{-1}	1.8	1.80 ± 0.04	2.0	100

[a] Mean and confidence interval (P = 0.95)

Table S6 The pre-treatment processes performed for real samples

Dye	Sample	Dilution	Filtration	Dye content in the analyzed sample, mg L^{-1}	*t* and *F* values, estimated at a 95% confidence level[a] for determination after extraction by both[b] ABSs
Azorubine	Energy carbonated drink	1:100	–	47 ± 2	0.68; 0.4
	Highly carbonated strawberry drink	1:100	–	32 ± 2	2.37; 2.25
	Cotton candy flavored drink	1:10	+ through nonwoven polypropylene	2.4 ± 0.2	2.5; 8.9
	Mini fruit jelly "Cherry flavor"	1.500 g of sample dissolved in 50.0 mL of distilled water at moderate heat (up to 70 °C)	+ through nonwoven polypropylene	29.0 ± 1.5[c]	2.32; 2.25
	Candy	1.500 g of sample dissolved in 50.0 mL of distilled water at moderate heat (up to 70 °C)	+ through nonwoven polypropylene	18.3 ± 0.5[c]	1.93; 4.05
Allura Red	Strawberry-cream carbonated drink	1:100	–	18 ± 2	–
	Strawberry-flavored carbonated soft drink	1:100	–	73 ± 4	–
	Vitamin strawberry-vanilla drink/	1:10	–	10.5 ± 0.5	–
	Mini fruit jelly "Peach flavor"	1.500 g of sample dissolved in 50.0 mL of distilled water at moderate heat (up to 70 °C)	+ through nonwoven polypropylene.	5.5 ± 0.5[c]	–

Sunset Yellow	Vitamin mango and kiwi drink	1:10	+ through nonwoven polypropylene	2.8 ± 0.2	–
	Orange-flavored carbonated drink	1:100	+ through nonwoven polypropylene	36 ± 2	–
	Mini fruit jelly"Passion flavor"	1.500 g of sample dissolved in 50.0 mL of distilled water at moderate heat (up to 70 °C)	+ through nonwoven polypropylene	11.5 ± 0.5[c]	–
Fast Green	Mouthwash “Listerine”	1:25	–	3.8 ± 0.3	–
Tartrazine	Carbonated drink with cream flavor	1:10	+ through nonwoven polypropylene	2.8 ± 0.4	–
	Carbonated drink based on flavors “Mountain Dew”	1:10	–	6.3 ± 0.6	–

[a] $t_{critical} = 2.78$, $F_{critical} = 19.2$

[b] TBABr–K_3Cit–H_2O, TBABr–KSCN–H_2O

[c] μg g^{-1}

Table S7 Data for plotting the TBABr–KSCN–H_2O diagram

Stock solutions, mol L^{-1}		Volume, mL			Concentrations at the end points of titration**, mol L^{-1}		c_{TBA} / c_{KSCN}	c_{TBA} + c_{KSCN}	c_{KSCN} / c_{TBA}
c_{TBA}	c_{KSCN}	TBABr	KSCN	H_2O	$c_{TBABr, e.p.t.}$	$c_{KSCN. e.p.t.}$			
1.5001	1.0000	5.00	2.90*	0.00	0.9494	0.3671	2.59	1.3165	0.39
1.5001	1.0000	3.00	1.50	2.00	0.6924	0.2308	3.00	0.9231	0.33
1.5001	1.0000	2.00	0.95	2.00	0.6061	0.1919	3.16	0.7980	0.32
0.5001	0.5002	10.00	3.50	0.00	0.3704	0.1297	2.86	0.5001	0.35
0.5001	0.5002	10.00	4.30	4.00	0.2733	0.1175	2.33	0.3908	0.43
0.5001	0.5002	10.00	5.60	8.00	0.2119	0.1187	1.79	0.3306	0.56
0.5001	0.5002	10.00	7.60	12.00	0.1690	0.1284	1.32	0.2974	0.76
0.5001	0.5002	5.00	5.50	8.00	0.1352	0.1487	0.91	0.2839	1.10
0.5001	0.5002	5.60*	5.00	8.00	0.1506	0.1345	1.12	0.2850	0.89
0.5001	0.5002	6.10	10.00	12.00	0.1086	0.1780	0.61	0.2866	1.64
0.5001	0.5002	3.90	10.00	8.00	0.0891	0.2284	0.39	0.3175	2.56
0.5001	0.5002	2.40	10.00	4.00	0.0732	0.3050	0.24	0.3782	4.17
0.5001	0.5002	1.52	10.00	0.00	0.0660	0.4342	0.15	0.5002	6.58
0.5001	1.0000	1.00	5.00	2.00	0.0625	0.6250	0.10	0.6875	10.00
0.5001	1.0000	0.70	5.00	0.00	0.0614	0.8772	0.07	0.9386	14.28
0.5001	2.0001	0.95	5.00	2.00	0.0598	1.2579	0.05	1.3177	21.05
0.5001	2.0001	0.72	5.00	0.00	0.0629	1.7483	0.04	1.8113	27.77

* the cell shaded in gray corresponds to the volume of the titrant

$$^{**}c_{KSCN,\text{e.p.t.}} = \frac{c_{\text{KSCN},\ stock\ solution} \cdot V_{\text{KSCN}}}{V_{\text{KSCN}} + V_{\text{TBABr}} + V_{\text{H}_2\text{O}}};\ c_{\text{TBABr,e.p.t.}} = \frac{c_{\text{TBABr},stock\ solution} \cdot V_{\text{TBABr}}}{V_{KSCN} + V_{\text{TBABr}} + V_{\text{H}_2\text{O}}}.$$

Table S8 Data for plotting the TBABr–K_3Cit–H_2O diagram

Stock solutions, mol L^{-1}		Volume, mL			Concentrations at the end points of titration**, mol L^{-1}		c_{K3Cit}/c_{TBABr}
c_{TBABr}	c_{K3Cit}	K_3Cit	TBABr	H_2O	$c_{K3Cit, e.p.t.}$	$c_{TBABr, e.p.t.}$	
1.000	2.5700	2.50	7.50	0.70*	0.6005	0.7009	0.86
		2.70	7.30	1.60	0.5982	0.6293	0.95
		3.00	7.00	2.90	0.5977	0.5426	1.10
		3.30	6.70	3.60	0.6236	0.4926	1.27
		4.00	6.00	4.65	0.7017	0.4096	1.71
		4.30	5.70	5.10	0.7319	0.3775	1.94
		5.00	5.00	5.85	0.8107	0.3155	2.57
		5.30	4.70	6.20	0.8408	0.2901	2.90
		6.00	4.00	6.62	0.9278	0.2407	3.86
		6.30	3.70	6.90	0.9580	0.2189	4.38
		7.00	3.00	7.30	1.0399	0.1734	6.00
		7.30	2.70	7.10	1.0971	0.1579	6.95
		8.00	2.00	7.50	1.1749	0.1143	10.28
		8.30	1.70	7.30	1.2330	0.0983	12.55
		8.50	1.50	7.20	1.2701	0.0872	14.56
		8.70	1.30	6.90	1.3230	0.0769	17.20
		9.00	1.00	6.30	1.4190	0.0613	23.13
		9.50	0.50	5.20	1.6063	0.0329	48.83

* the cell shaded in gray corresponds to the volume of the titrant

** $c_{K_3Cit,e.p.t.} = \frac{c_{K_3Cit,\ stock\ solution} \cdot V_{K_3Cit}}{V_{K_3Cit} + V_{TBABr} + V_{H_2O}}$; $c_{TBABr,e.p.t.} = \frac{c_{TBABr,stock\ solution} \cdot V_{TBABr}}{V_{K_3Cit} + V_{TBABr} + V_{H_2O}}$.

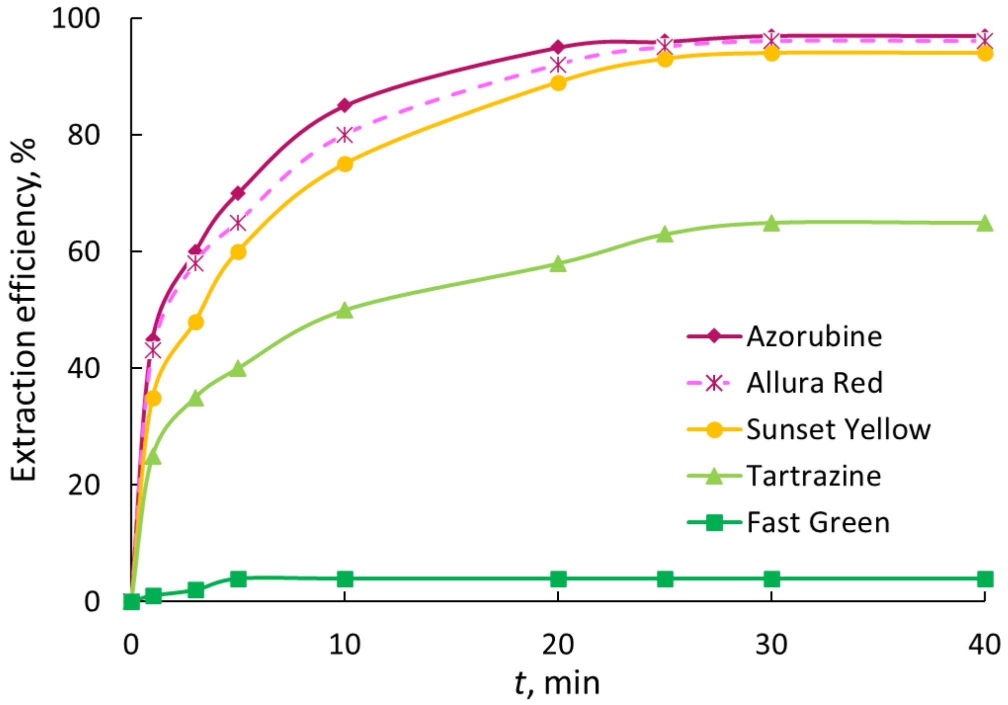

Fig. S1 Effect of phase contact time on dyes extraction efficiency in 1-octanol–H_2O system. Dyes concentration: Azorubine, Allura Red, Sunset Yellow, Tartrazine, $4.0{\cdot}10^{-5}$ mol L^{-1}; Fast Green, $8.0{\cdot}10^{-6}$ mol L^{-1}; c_{HCl} = 5 mol L^{-1}; $V_{aq.phase} = V_{octanol}$ = 2.0 mL.

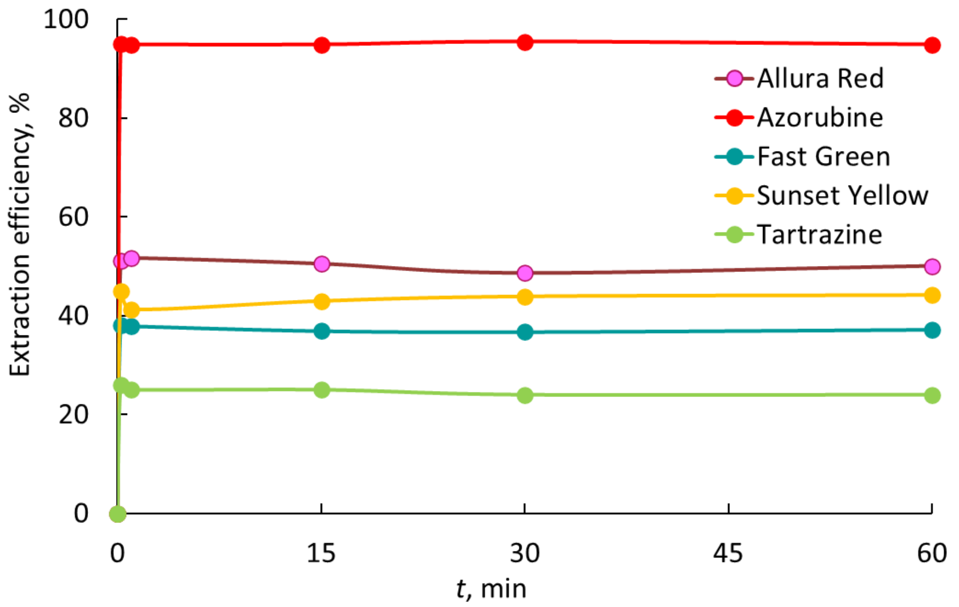

Fig. S2 Effect of phase contact time on dyes extraction efficiency in TBABr–KSCN–H_2O ABS. $c_{TBABr} = c_{KSCN} = 0.2$ mol·L^{-1}, $V_{lower\ phase} = 3.8$ mL and $V_{upper\ phase} = 0.09$ mL, 0.1 mol·L^{-1} HCl. Dyes concentration: Azorubine, Allura Red, Sunset Yellow, Tartrazine, 25 mg L^{-1}; Fast Green, 8 mg L^{-1}.

Chromatographic procedure for determination of Br^- and SCN^-. An ICS Dionex-2100 apparatus with an 100 mm × 4.0 mm i.d. stainless steel chromatographic column packed with polymer-based anion exchanger EBPEI-GI 2, flow rate 1.0 mL/min, gradient elution mode was used. Poly(styrene-divinylbenzene)-based anion exchanger EBPEI-GI 2 was employed as the stationary phase, which synthesis procedure and properties discussed in detail [1]. The gradient elution mode was employed: 0–9 min — 2 mM KOH, 9–9.1 min — 2–40 mM KOH. Suppressor current: 82 mA. Analysis time with reconditioning for initial state: 21 min.

References

1. A.V. Gorbovskaia, E.K. Popkova, A.S. Uzhel, O.A. Shpigun, Mixed-Mode Polymer Stationary Phases of Increased Hydrophilicity with Grafted Polyethyleneimine and Polyglycidol, J. Anal. Chem. 79 (2024) 464–475. https://doi.org/10.1134/S1061934824040063.
